# A Case of Periosteal Sarcoma

**Published:** 1910-02-19

**Authors:** 


### Surgery.

### A CASE OF PERIOSTEAL SARCOMA.

The patient was a man aged forty-five, who complained chiefly of pain and swelling in the left arm and in the left buttock, and of swelling of the abdomen. His family history was vague, and seemed to have no bearing on the case. The patient himself had had gonorrhœa as a young man, but had never had a chancre, and had always enjoyed good health. Three months previous to the consultation the man fell down upon his left arm, striking it violently upon a hard pavement, but not breaking the bone. The injury caused him much pain at the time, and shortly afterwards the arm swelled gradually and the pain increased. Up to three weeks ago the man continued with his work, which did not entail the use of the left arm. A week after this, pain began in the left buttock, running down the left leg posteriorly, and also involving the left groin. During the last few days this pain in the left leg prevented standing. Four days ago the patient first noticed that his abdomen was swelling. There was no abdominal pain, but a disagreeable sense of discomfort after taking food.

The temperature was 98.2° F., the pulse rate 100 per minute, and the respiration rate 20. The man presented an unhealthy appearance. His face was flushed and drawn with pain. His limbs were thin, and his mucous membranes anæmic. There was a large swelling which involved the greater part of the shaft of the left humerus. It was about five inches in length, occupying chiefly the anterior and inner aspect of the bone. It was irregular, nodulated, and gave the impression of fluctuation in parts. It was painful, the pain being increased by any movement of the arm. It was tender on firm pressure. There was some reddening of the skin over it, with slight œdema, and small congested venules were scattered about over the man. The stretching of the skin made it rather shiny. There was much loss of power in the left arm and hand. No crepitus could be obtained. The measurements of the arm over the mass were 11½ inches above, 12¼ inches over its middle, and 12 inches at its lower end; the circumference of the other arm was 7½ inches. There was no bruit, no thrill, and no expansile pulsation in the lump. The tissues showed no discoloration suggestive of hæmorrhage into the part. The joints above and below were normal. A small, hard, tender gland could be felt in the left axilla; there were no glands noticeable in the right. The left forearm was wasted, but not more so than the right, the condition of both appearing to be due to the patient's general condition and cachexia.

There was an extensive and diffuse swelling over the left ilium, beginning about two inches below the iliac crest, and extending over an area of about five square inches. It was of irregular shape. The skin over it was hot to the touch, but otherwise normal. The mass was both tender and painful. It was hard, and firmly attached to the bone. It appeared to extend into the pelvis through the sciatic notch. The abdomen was distended; resonant around the umbilicus, but dull in the flanks, the dulness shifting as the patient's position was changed. The liver could be felt coming three inches below the costal margin in the nipple line. It was tender, and was thought to be nodulated. There was slight œdema of the feet. There were small, hard, tender glands in the posterior triangle of the neck on each side. The heart seemed to be normal, though the pulse rate was 100. There was a leucocytosis of 14,500 white corpuscles per cub. mm. of blood; and the differential count showed 5 per cent. of coarsely granular eosinophile corpuscles. The chest moved well with respiration, and the lung signs were natural, except for slight deficiency of vesicular murmur at the right base posteriorly, where a few non-consonating râles could also be heard. The testicles and spermatic cords seemed natural. The urine had a specific gravity of 1,026; it was acid, and of a high colour, and it gave an abundant precipitate of urates on cooling. Neither albumen nor sugar was present in it.

The patient died less than a fortnight after the consultation. The condition turned out to be a small round celled periosteal sarcoma, primary on the left humerus, with secondary sarcomatous deposits in the lungs, liver, axillary, cervical, mediastinal and abdominal lymphatic glands, in the pelvis, and in the periosteum of the left os innominatum.

One point of interest in the case lies in the question of whether the injury had anything to do with originating the primary growth in the injured arm. It is very possible that the conjunction of the injury with the growth in the same site immediately afterwards was a mere coincidence. This is an old problem in pathology, but it is one which assumes a very important aspect in connection with the various Acts related to Workmen's Compensation.

